# External validation of a predictive model for post-treatment persistent disease by ^131^I whole-body scintigraphy in patients with differentiated thyroid cancer

**DOI:** 10.1007/s00259-025-07124-2

**Published:** 2025-02-25

**Authors:** Carmela Nappi, Rosario Megna, Emilia Zampella, Fabio Volpe, Leandra Piscopo, Maria Falzarano, Carlo Vallone, Leonardo Pace, Mario Petretta, Alberto Cuocolo, Michele Klain

**Affiliations:** 1 Department of Advanced Biomedical Sciences, University of Naples Federico II, Naples, Italy; 2 Institute of Biostructures and Bioimaging, CNR, Naples, Italy; 3 Department of Medicine, Surgery and Dentistry, University of Salerno, Salerno, Italy; 4 IRCCS Synlab SDN, via Emanuele Gianturco 113, 80143 Naples, Italy

**Keywords:** Thyroid cancer, Whole-body scintigraphy, Risk analysis, External validation

## Abstract

**Purpose:**

We performed an external validation of a predictive model for persistent/metastatic disease in patients with differentiated thyroid cancer (DTC) at post-treatment ^131^I whole-body scintigraphy (WBS).

**Methods:**

Our study population included 836 patients (median age 44 years, 78% women) with DTC referred from 1994 to 2021 at our center. Age, sex, histology, T stage, N stage, American Thyroid Association risk classes, thyroid-stimulating hormone, radioactive iodine (RAI) activity, and thyroglobulin (Tg) levels were considered potential predictors of post-treatment WBS results. For the external validation, N stage and Tg levels were put into the decision tree (DT) model using its same Tg cut-off values.

**Results:**

Ninety-nine patients (12%) had positive post-treatment WBS. The area under receiver operating characteristic (ROC) curve for predicting WBS findings through the external validation was 0.60 (95% confidence interval, CI, 0.56–0.64), and positive and negative predictive values were 58% (95% CI, 41–74%) and 90% (95% CI, 88–92%). We also developed an internal model including the independent predictors of WBS findings (i.e., Tg levels, T stage, N stage, and RAI activity). For this model the area under ROC curve was 0.75 (95% CI, 0.69–0.81), and positive and negative predictive values were 90% (95% CI, 68–99% and 88–92%).

**Conclusions:**

The external validation of the proposed DT model has a limited value for predicting post-treatment ^131^I-WBS findings in our patients. The internal model including also T stage and RAI activity demonstrates higher predictive value.

## Introduction

Although management of differentiated thyroid cancer (DTC) benefits from a large body of evidences guided by updated guidelines [1–4], there is still an open debate on the best method to predict persistent and/or metastatic disease after surgical treatment and to achieve the best treatment strategy in patients with DTC referred to ^131^I therapy. Indeed, DTC forms with an excellent prognosis, especially when detected early, need less aggressive treatment strategies, including de-escalation of therapy in treatment decisions and the activities eventually administered. Conversely, in addition to high dose ^131^I therapy as initial approach, patients with advanced thyroid cancer benefit from more aggressive therapies such as localized treatments, targeted drugs against vascular endothelial growth factor receptor, mitogen-activated protein kinase pathway-specific therapies, gene rearrangements or fusions, and immunotherapy [5–7]. In patients with DTC undergoing radioactive iodine (RAI) therapy after surgery, the post-treatment whole-body scintigraphy (WBS) is considered the method of choice to identify ^131^I avid local or distant metastasis. Recently, Giovanella et al. [8] proposed a decision tree (DT) model for predicting post-treatment WBS results developed considering 1314 patients with DTC enrolled in 5 European centers [8]. This model included lymph node (N) stage and thyroglobulin (Tg) levels as independent variables founding the first significant split for the N variable and 3 significant decision nodes for Tg values of 35.0, 7.1, and 23.3 ng/mL. In the present study, we performed an external validation of the prediction model by Giovanella et al. [8] to evaluate its ability for predicting persistent disease in patients with DTC using data from our center.

## Methods

### Patients

The study population included patients with DTC referred to our academic center for RAI therapy after surgery from 1994 to 2021. Consecutive patients 18 years and older with histologically proved DTC who underwent (near-)/total thyroidectomy and thyroid hormone withdrawal assisted ^131^I therapy were included. American Joint Committee on Cancer 7th edition classification was used for TNM staging [2]. Patients were categorized in the low, intermediate and high-risk group according to the American Thyroid Association (ATA) guidelines and practice recommendations [2]. Serum Tg, Tg-antibody, and thyroid-stimulating hormone (TSH) levels were obtained after withdrawal of L-thyroxine treatment, one day before RAI administration [9]. Patients without information on Tg-antibody levels, pre-ablation TSH, Tg, and post-treatment WBS results were excluded from the study.

### Radioiodine treatment

Low iodine diet was recommended for 7–14 days before ^131^I administration: patients were asked to discontinue the use of iodine containing preparations and medications for 3 weeks, and no water-soluble iodinated contrast medium was administered at least for 6 weeks before RAI treatment. Thyroid hormone (L-thyroxine) treatment was withdrawn for 3 weeks before RAI administration and until serum TSH reached an arbitrary level of 30 mIU/L; in patients with a TSH below this level, withdrawal was prolonged for 7 days and TSH level was measured again before RAI administration. Patients received written information concerning RAI treatment and radioprotection before oral administration of ^131^I as capsules. Patients selected for RAI administration underwent thyroid hormone withdrawal and received a ^131^I activity determined empirically based on the individual clinical characteristics according to the ATA guidelines [2]. The administered dose ranged from 30 to 50 mCi in low-risk patients, from > 50 to 100 mCi in intermediate risk patients and from > 100 to 200 mCi in high risk patients. Radioprotection issues were managed in strict adherence to national regulations as previously described [10]. Patients were hospitalized for 2.5–3 days in protected rooms. Abundant hydration and laxative treatment were given during the hospitalization.

### Post-treatment WBS

Post-treatment WBS was performed 7 days after ^131^I administration using a dual-head gamma camera equipped with thick crystals and high-energy collimators following the guidelines of the Society of Nuclear Medicine and Molecular Imaging/European Association of Nuclear Medicine [11]. WBS were classified as negative (i.e., absent uptake within the thyroid bed and absent non-physiological uptakes in other regions, or uptake within the thyroid bed without non-physiological uptakes in other regions) or positive (pathological uptake outside the thyroid bed, with or without uptake within the thyroid bed) [8]. In presence of ^131^I uptake in the neck region, planar imaging was integrated with single photon emission computed tomography (SPECT) and ultrasound to determine if the WBS activity was likely due to DTC secondary foci or remnant thyroid tissue. In case of increased uptake outside thyroid bed, patients were referred to CT imaging to better localize the pathological lesion.

### Statistical analysis

Continuous data are expressed as median and interquartile range (IQR), and categorical data as percentage. Differences between groups were analyzed by the non-parametric unpaired Wilcoxon signed-rank test or chi square test, as appropriate. Two-sided P-values < 0.05 were considered statistically significant. For the external validation, we considered the same DT software procedure as reported in [8] (*ctree* function, developed in the *party* R package), and included the N stage variable and the cut-off values of 7.1, 23.3, and 35.0 ng/mL for the Tg levels. We also developed an internal model considering a multivariable logistic regression (MLR) algorithm. Age, sex, histology, T stage (the size of the cancer and whether it has spread to nearby structures), N stage (spread of cancer to nearby lymph nodes), ATA risk classes, TSH, RAI activity, and Tg levels were identified as potential predictors and were put into MLR model for predicting post-treatment WBS results. For constructing the internal model, we implemented the following three steps: 1) a first MLR containing all potential predictors was performed. Then, the covariates with a P-value > 0.10 (age, sex, histology, risk classes, and TSH) were excluded from the model; 2) a second MLR, containing T stage, N stage, RAI activity, and Tg levels as covariates was performed. At this step, all the variables were found to be significant statistically (P-value < 0.05) and were included in the model. To minimize overfitting, the10-fold cross-validation method was used; 3) we attempted to reduce the dimensionality of the model while maintaining or improving model performance. Then, considering the 4 combinations with 3 covariates, we performed the MLR again as at point 2). In all tested cases, the model performance decreased and the model with 4 covariates was adopted to develop a nomogram.

The performance of the external validation and internal model was obtained using the same metrics as in [8]: accuracy, positive predictive values (PPV), negative predictive values (NPV), and area under receiver operating characteristic (ROC) curve. We assessed the accuracy of probabilistic predictions obtained with the MLR algorithm also by Brier score. Confidence intervals (CI) were computed at 95%. CI related to area under ROC curves and Brier score were computed by 1000 bootstrap resampling. The statistical analysis and models were performed using R version 4.3.0 (The R Foundation for Statistical Software, Vienna, Austria), and *dplyr*, *caret*, *party, DescTools, ggplot2*, and *pROC* extra packages.

## Results

Clinical characteristics of study population according to post-treatment WBS findings are shown in Table 1. The study population included 836 patients (median age 44 years, 78% women). In the entire cohort, median of Tg levels measured under withdrawal form LT4 administration, previous to RAI administration, was 5.1 ng/mL. The high-risk group included T4 stage (*n* = 40), N1 with the largest lymph node metastasis > 3 cm (*n* = 36), presence of metastases (*n* = 24), post-surgery Tg levels indicative of distant metastases (*n* = 148), and follicular thyroid cancer with > 4 foci of vascular invasion (*n* = 16). A total of 99 (12%) patients had positive post-treatment WBS. Among these patients, 14 had only local persistent disease, 25 local and distant metastases, and 60 only distant metastases. Of note, 20 patients had known distant metastases at diagnosis.

**Table 1:** Demographic, clinical, and pathological characteristics of study population according to whole body scintigraphy findings

	All patients (*n* = 836)	Negative WBS (*n* = 737)	Positive WBS (*n* = 99)	*P*-value
Age (years)	44 (33–45)	43 (34–55)	44 (33–60)	0.37
> 55 years, *n* (%)	197 (24)	166 (23)	31 (31)	0.07
Female gender, *n* (%)	652 (78)	584 (79)	68 (69)	< 0.05
Ioduria value (mg/dL)	55 (9–94)	60 (16–109)	55 (9–92)	0.16
TSH (mUI/mL)	60 (37–93)	60 (37–93)	60 (36–95)	0.93
Tg (ng/mL)	5.1 (1.1–18.1)	4.8 (1.0–15.0)	23.8 (2.7–202)	< 0.001
Histology				< 0.001
PTC, *n* (%)	431 (52)	379 (52)	52 (53)	
PTC-V, *n* (%)	273 (33)	241 (33)	32 (32)	
FTC, *n* (%)	132 (16)	117 (16)	15 (15)	
ATA risk				< 0.001
Low, *n* (%)	386 (46)	354 (48)	32 (32)	
Intermediate, *n* (%)	186 (22)	164 (22)	22 (22)	
High, *n* (%)	264 (32)	219 (30)	45 (46)	
T stage				< 0.001
1, *n* (%)	452 (54)	418 (57)	34 (34)	
2, *n* (%)	234 (28)	205 (28)	29 (29)	
3, *n* (%)	110 (13)	87 (12)	23 (23)	
4, *n* (%)	40 (5)	27 (4)	13 (13)	
N stage				< 0.001
0, *n* (%)	693 (83)	629 (85)	64 (65)	
1, *n* (%)	143 (17)	108 (15)	35 (35)	
2, *n* (%)	0 (0)	0 (0)	0 (0)	

Values are expressed as median (interquartile range) or as number (percentage) of subjects; *PTC* papillary thyroid cancer, *FTC* follicular thyroid cancer, *PTC-V* variant of papillary thyroid cancer, *ATA* American Thyroid Association

Mean administered RAI activity was 97.3 ± 27.0 mCi in the entire study population, and it was 93.6 ± 23.7 mCi in patients with negative and 117.8 ± 38.8 mCi in those with positive (*P* < 0.001) post-treatment WBS.

**Fig. 1:**
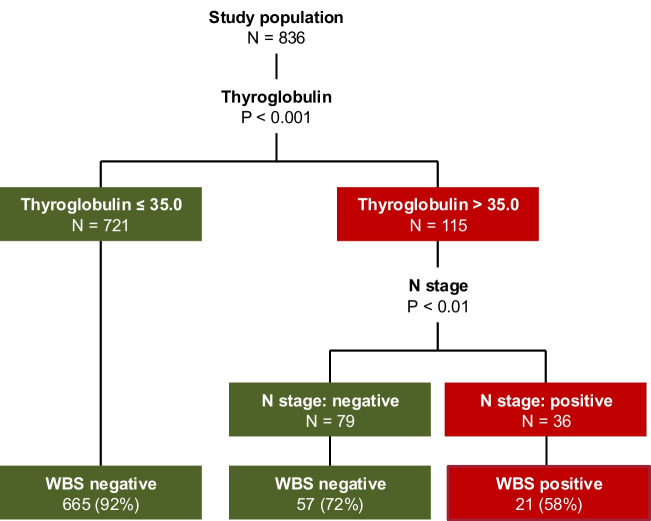
Graphical representation of classification tree according to the model presented in [8]. In our cohort, Tg values subdivides the population into two subgroups. In case of Tg > 35 ng/mL, the probability to have persistent/metastatic disease was 58% in patients with lymph node involvement and 28% in those without lymph node involvement. The probability of persistent/metastatic disease decreased to 8% in patients with Tg ≤ 35 ng/mL

Figure 1 shows the validation according to the DT prediction of the external model. In our cohort, we obtained a DT with the first ramification at Tg cut-off of 35.0 ng/mL. No further ramification was found for patients with Tg below this value. For patients with Tg values above 35.0 ng/mL, N stage determined a second and last split into two branches. The Tg cut-off at 7.1 and 23.3 ng/mL were not significant in our cohort.

**Fig. 2:**
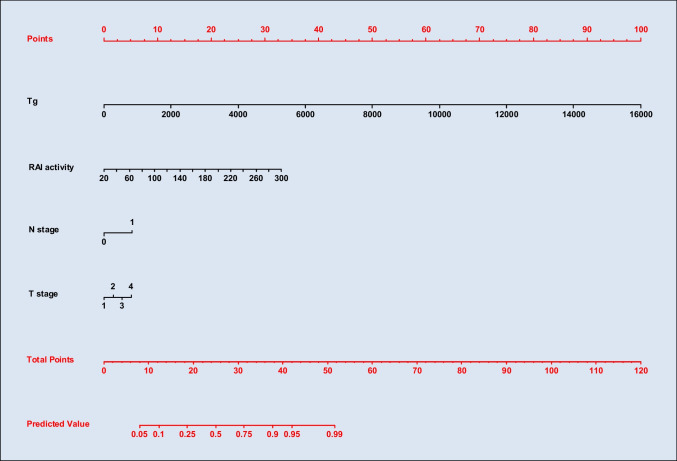
Nomogram derived from the internal model developed by the multivariable logistic regression (MLR) algorithm. The risk to have a positive post-treatment WBS is calculable summing *points* associated to the four covariates as *total point* and comparing it with the *predicted value*

Table 2 summarizes the internal model results obtained using the MLR algorithm. More significant predictors were ^131^I activity and N stage (*P* < 0.001 for both), followed by Tg levels and T stage (*P* < 0.05 for both). The nomogram derived from this model is shown in Fig. 2. The risk to have a positive post-treatment WBS is calculable summing *points* associated to the four variables as *total point* and comparing it with the *predicted value*. A predicted risk value greater than 5% is expected in patients with a total point from 8 onwards. For this model, we obtained a Brier score of 0.0841 indicating good accuracy of probabilistic predictions.

**Table 2:** Multivariable logistic regression for MACE

	Coefficient	Standard error	*P*-value	Odds ratio (95% CI)
Intercept	−5.024	0.555	< 0.001	−
Thyroglobulin	0.0011	0.0004	< 0.05	1.001 (1.000, 1.002)
^131^I activity	0.020	0.005	< 0.001	1.02 (1.01, 1.03)
N stage	0.914	0.257	< 0.001	2.49 (1.50, 4.10)
T stage	0.291	0.126	< 0.05	1.34 (1.04, 1.71)

**Fig. 3:**
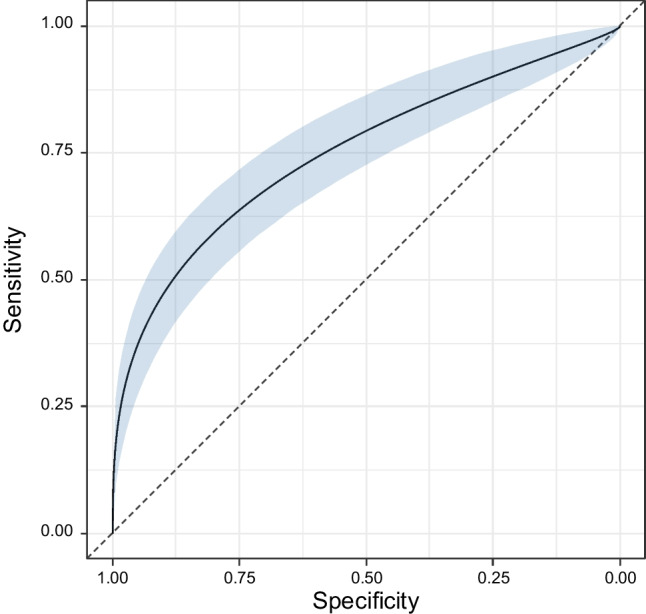
Receiver operating characteristic (ROC) curve related to the internal model obtained by the multivariable logistic regression (MLR) algorithm. The area under ROC curve is 75% (95% CI, 68–81%), and the Youden's index is (0.811, 0.583)

**Fig. 4:**
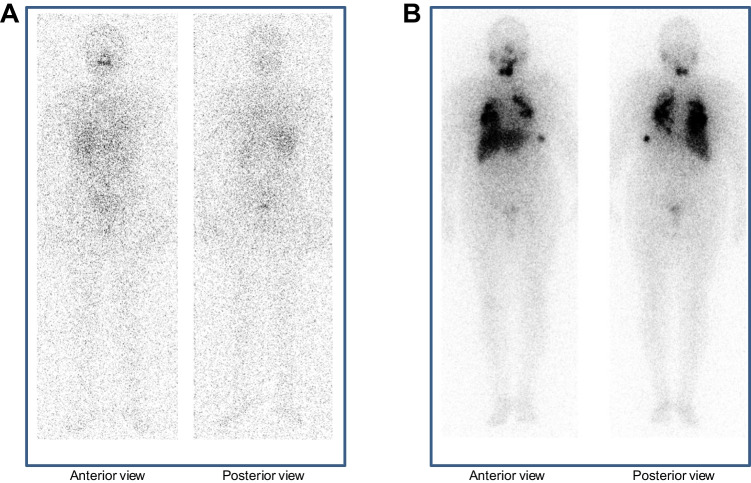
WBS images related two patients, whose data were used for the risk calculation by our nomogram. Patient A (negative WBS) resulted to have total points of 10 and a predicted risk value of 7% for positive WBS. Patient B (positive WBS) resulted to have total points of 111 and a predicted risk value > 99% for positive WBS

Table 3 reports the predictive values, accuracy and overall performance for the external validation and internal model. The area under ROC curve for the external validation was 0.60, and PPV and NPV were 58% and 90%. For the internal model the area under ROC curve was 0.75, and PPV and NPV were 90%. Figure 3 shows the ROC curve with the 95% CI for the internal model. Figure 4 shows the WBS images in two patients, whose data were used as examples for the risk calculation by our nomogram. Patient A is a 39 year-old woman, found to be a negative post-treatment WBS. Tg value was 0.1 ng/mL (points = 0), RAI activity = 108 mCi (points = 10), N stage = 0 (points = 0), and T stage = 1 (points = 0), with a predicted risk value of 7% for positive WBS. Patient B is a 76 year-old woman, resulted to be a positive post-treatment WBS. Tg value was 12,000 ng/mL (points = 75), RAI activity = 231 mCi (points = 25), N stage = 1 (points = 6), and T stage = 3 (points = 5), with a total points of 111 and a predicted risk value greater than 99% for positive WBS.

**Table 3:** Predictive values, accuracy and overall performance for the external validation and internal model

	External validation	Internal model
PPV (95% CI)	58% (41–74)	90% (68–99)
NPV (95% CI)	90% (88–92)	90% (88–92)
Accuracy (95% CI)	75% (0.69–0.81)	90% (88–92)
AUROC (95% CI)	0.60 (0.56–0.64)	0.75 (0.69–0.81)

*PPV* positive predictive value, *NPV* negative predictive value, *AUROC* area under receiver operating characteristic curve

## Discussion

To identify persistent/metastatic disease in patients with DTC, the present study has externally validated the ability of a previously developed prediction model [8] and realized an internal model. In the last years, external validation studies have been carried out in several clinical fields [12–16], to assess the generalization in other populations of predictive risk models. The algorithm proposed by Giovanella et al. showed an accuracy of 88%, a PPV of 68%, and a NPV of 90% for the training data set (70% of the overall study population). Lower PPV (60%), and similar NPV and accuracy were observed for the test data set (remaining 30% of the overall study population). An area under ROC curve of 0.68 was obtained on the overall study population [8].

Prior to our study, the proposed model [8] was validated by data from a cohort of 240 patients [17]. In that validation, the following metric values were achieved: PPV of 29%, NPV of 91%, and accuracy of 87%. The authors declared that lower PPV can be explained partially by the lower proportion of positive post-treatment WBS (10.4%) compared with most centers (from 6.0 to 20.5% for the 5 centers; overall 15.2%) reported in the proposed model [8]. As showed by our validation, the tested DT model [8] demonstrated a limited value for predicting a positive post-treatment WBS also in our cohort. Similarly to the study by Sasaki et al. [16], the prevalence of positive post-treatment WBS among our patients was lower than those observed by Giovanella et al. [8] (11.8% vs. 15.2%). If on the one hand we agree that the lower PPV can be explained partially by the prevalence of positives, on the other hand it is necessary to consider that patients’ clinical characteristics were different between the two cohorts. For example, patients in our cohort were younger, with higher Tg levels, and different histology and T stage percentages compared to patients studied by Giovanella et al. [8]. Instead, other variables such as gender and N stage percentages were similar. Likely, the DT model could have a better performance with a cohort containing a greater prevalence of positive patients. On this topic, from the point of view of machine learning algorithms, another non-negligible consideration is also required. In fact, it is necessary to consider that these procedures tend to classify as negative a patient if most of dataset contains negative patients. Therefore, also this intrinsic characteristic related to machine learning can explained partially the high NPV values observed. Anyhow, it is known that a prediction model performs best in the population it was derived from.

The less than optimal results obtained by our external validation led us to develop an internal model based on another predictive algorithm, considering more variables. In addition to Tg levels and N stage variables, also T stage and RAI activity showed an independent value in predicting positive post-treatment WBS. This finding is not surprising for two main reasons. The probability to obtain a positive WBS with higher administered RAI activities is consistent with the decision to administer higher RAI doses in patients with greater tumor burden. Indeed, RAI activity to administer is determined on several factors including the presence of metastatic disease [18, 19]. Although, to our knowledge there are no robust data demonstrating the effectiveness of higher RAI activities such as 200 mCi as compared to lower activities, RAI dose selection is still an open ground of debate. This is even more relevant on the light of recent advances on dosimetric approaches taking into account individual specific pharmacokinetics and calculation of specific absorbed doses for macroscopic and measurable lesions resulting in higher doses for greater tumor burden [20]. Thus, a reasonable doubt concerns the concept that if it is true that the more you see, the more you treat, it is also true that the more you give, the more you may see. However, this is still object of an open question taking into account, over clinical and dosimetry considerations also the presence of tumor heterogeneity. Although there is a potential interplay between RAI activity and ATA risk categories, given that patients at high-risk usually receive higher doses, when put together into the internal model only RAI activity demonstrated a predictive value for WBS positivity. This finding is consistent with increased evidence that further parameters should be considered for risk assessment, such as oncogenic genetic alterations, beyond those incorporated into the 2015 ATA risk classification [21].

The internal model based on MLR showed an increase of the area under ROC curve and a better classification of post-treatment WBS than external validation. In other words, adaptation of the algorithm to the study population improved the predictions. The logistic model was particularly performing with respect to positive patients, showing a very similar predictive value for PPV and NPV and limited 95% CI. These are important characteristics to obtain an efficient test to classify negative as well as positive patients. Notably that the MLR algorithm uses continuous variables without the need for cut values as required in the DT algorithm, and this allows us to calculate the risk as a continuous value and avoid other problems caused by categorization. On this topic, some considerations are reported in the following. Categorization assumes that the relationship between predictors and outcome is flat within intervals, and this hypothesis is far less reasonable than a linearity assumption in most cases. Furthermore, categorization assumes a cutoff in response as interval boundaries are crossed. Lastly, optimal cut points do not replicate over studies, as demonstrated in our external validation [22].

The limitation of the present study was due to the relatively small number of patients. In addition, our patient population came from only one center. This characteristic makes our data sample more homogeneous and less influenced by random factors. Although the internal model without cut-offs for continuous variables could be more adaptable than the Giovanella's model, it would be desirable to carry out external validation to verify its performance and adaptability in different populations.

## Conclusions

The previously proposed DT model based on N stage and Tg levels had a limited value for predicting a positive post-treatment ^131^I WBS in our validation cohort. The internal model, also including T stage and RAI activity, demonstrated a higher predictive value as compared to the external validation. Further studies are looked-for for a more accurate prediction in the field of post-treatment ^131^I WBS.

## Data Availability

The datasets generated during and/or analyzed during the current study are available from the corresponding author on reasonable request.
